# KUD773, a phenylthiazole derivative, displays anticancer activity in human hormone-refractory prostate cancers through inhibition of tubulin polymerization and anti-Aurora A activity

**DOI:** 10.1186/s12929-014-0107-x

**Published:** 2015-01-07

**Authors:** Chia-Chun Yu, Shih-Ping Liu, Jui-Ling Hsu, John TA Hsu, Konstantin V Kudryavtsev, Jih-Hwa Guh

**Affiliations:** School of Pharmacy, National Taiwan University, Taipei, Taiwan; Department of Urology, National Taiwan University Hospital, Taipei, Taiwan; Institute of Biotechnology and Pharmaceutical Research, National Health Research Institutes, Zhunan, Taiwan; Department of Medicinal Chemistry, Faculty of Chemistry, M.V. Lomonosov Moscow State University, Moscow, Russian Federation; Institute of Physiologically Active Compounds, Russian Academy of Sciences, Moscow region, Russian Federation

**Keywords:** Hormone-refractory prostate cancer, Phenylthiazole derivative, Tubulin depolymerization, Aurora A kinase, Mitochondria-involved apoptosis

## Abstract

**Background:**

Hormone-refractory prostate cancer (HRPC), which is resistant to hormone therapy, is a major obstacle in clinical treatment. An approach to inhibit HRPC growth and ultimately to kill cancers is highly demanded.

**Results:**

KUD773 induced the anti-proliferative effect and subsequent apoptosis in PC-3 and DU-145 (two HRPC cell lines); whereas, it showed less active in normal prostate cells. Further examination showed that KUD773 inhibited tubulin polymerization and induced an increase of mitotic phosphoproteins and polo-like kinase 1 (PLK1) phosphorylation, indicating a mitotic arrest of the cell cycle through an anti-tubulin action. The kinase assay demonstrated that KUD773 inhibited Aurora A activity. KUD773 induced an increase of Cdk1 phosphorylation at Thr^161^ (a stimulatory phosphorylation site) and a decrease of phosphorylation at Tyr^15^ (an inhibitory phosphorylation site), suggesting the activation of Cdk1. The data were substantiated by an up-regulation of cyclin B1 (a Cdk1 partner). Furthermore, KUD773 induced the phosphorylation and subsequent down-regulation of Bcl-2 and activation of caspase cascades.

**Conclusions:**

The data suggest that KUD773 induces apoptotic signaling in a sequential manner. It inhibits tubulin polymerization associated with an anti-Aurora A activity, leading to Cdk1 activation and mitotic arrest of the cell cycle that *in turn* induces Bcl-2 degradation and a subsequent caspase activation in HRPCs.

## Background

The anticancer agents that act on different cellular targets may result in an arrest of the cell cycle at particular phase. Tubulin-targeting agents inhibit normal function of mitotic spindle, leading to a halt of the cell cycle at mitotic phase and a subsequent cell death [1-3]. Currently, tubulin-targeting agents have been successfully used in cancer chemotherapy against several types of cancers, including breast cancer, ovarian cancer and hormone-refractory prostate cancer (HRPC) [1,4,5]. However, the toxicities, such as peripheral neuropathy, myelosuppression and neutropenia, limit the effectiveness of tubulin-targeting agents [3,6]. Accordingly, a novel approach that reduces toxic effect but reserves the activity to trigger mitotic arrest and cell death can be helpful in cancer chemotherapy.

Aurora A kinase, belonging to a family of mitotic serine/threonine kinase, is implicated with critical processes in cell mitosis [7]. The regulation of Aurora A RNA, protein and kinase activity is dependent of cell cycle with peaking in the transition of G2 to mitotic phase [8]. Aurora A participates in centrosomal separation, formation of bipolar spindle and attachment of chromosomal kinetochore to mitotic spindle [8,9]. Several lines of evidence show that Aurora A is overexpressed in a wide variety of types of cancers, including hepatocellular carcinoma, prostate cancer, ovarian cancer and pancreatic cancer [10-12]. Frequent activation/overexpression of Aurora A in human primary prostate cancers has been reported. Furthermore, constitutive Aurora A activation displays higher oncogenic activity [13,14]. Activation/overexpression of Aurora A has been suggested to override mitotic spindle assembly checkpoint, leading to the resistance to taxol-induced apoptosis [3,15]. Moreover, it overrides cytotoxic agents-mediated down-regulation of Bcl-2 and Bcl-xL and decreased NF-κB activity [16,17], indicating that Aurora A plays a crucial role in addition to cell cycle regulation. These studies suggest that Aurora A can be a potential target for the development of cancer chemotherapeutic drugs.

HRPC occurs when hormone therapy fails to prevent the cancer growth for any longer. Advance prostate cancer often spreads to other parts of the body, including lymph nodes and bones. Therefore, HRPC is a treatment dilemma for practicing physician. Chemotherapy is one of the major choices for HRPC treatment. However, drug resistance developed in cancer cells is always a key concern in cancer chemotherapy. Imidazoles and thiazoles have been widely discovered as fundamental components of structurally diverse natural products that display a variety of activities, such as anticancer and antiviral activities [18-20]. The excellent pharmacological activities based on the azole skeletons have led to the generation of a lot of chemically synthetic compounds and have directed novel therapeutic applications [21]. To search for potential anticancer compounds against HRPCs, we have synthesized a series of derivatives based on thiazoles and imidazoles heterocycles. KUD773, 2-(1*H*-imidazol-1-yl)-4-(3-(trifluoromethyl)phenyl)thiazole [22], stood out and demonstrated anti-proliferative as well as apoptotic activities in PC-3 and DU-145, two HRPC cell lines. The study has characterized the anticancer activity and the mechanism of action of KUD773. The inhibition of tubulin polymerization and Aurora A activity has been delineated and the downstream signaling cascade has been studied in HRPCs.

## Methods

### Materials

**Figure 1:**
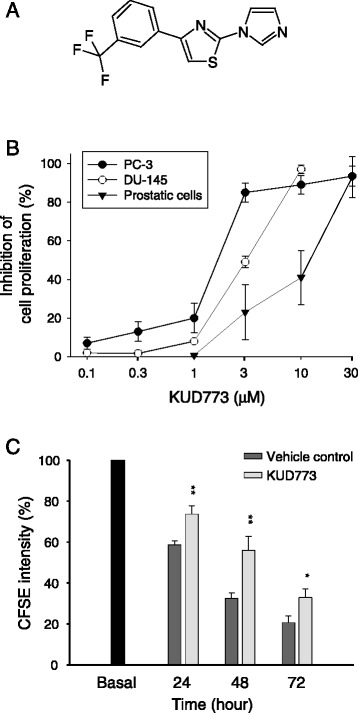
**Chemical structure of KUD773 and identification of anti-proliferative effect.** **(A)** Chemical structure of KUD773. **(B)** After the treatment with or without KUD773 for 48 h, the cells were fixed and stained with SRB and the data were analyzed. **(C)** PC-3 cells were labeled with CFSE and treated with vehicle or KUD773 (3 μM). The fluorescence intensity was determined by flow cytometric analysis. Data are expressed as mean ± SEM of three determinations. *P < 0.05 and **P < 0.01 compared with the respective control.

RPMI 1640 medium and fetal bovine serum (FBS) were obtained from GIBCO/BRL Life Technologies (Grand Island, NY). Antibodies to poly ADP ribose polymerase (PARP), caspase-7, Bcl-2, Bak, Bax, Bad, Mcl-1, and anti-mouse and anti-rabbit IgGs were obtained from Santa Cruz Biotechnology, Inc. (Santa Cruz, CA). Antibodies to actin, cyclin B1, cyclin A, caspase-9, caspase-8, phosphorylated cyclin dependent kinase-1 (cdk1) at Tyr15, p-cdk1^Thr161^, phosphorylated polo-like kinase 1 (PLK1) at Thr210, matrix metalloprotease-2 (MMP-2), MMP-9, p-histone H3^Ser10^, acetyl-histone H3, p-H2A.X^Ser139^ (γ-H2A.X) and GAPDH were from Cell Signaling Technologies (Boston, MA). The antibody to caspase-3 was from Imgenex, Corp. (San Diego, CA). The antibodies to β-tubulin and mitotic protein monoclonal 2 (MPM-2) were from BD Biosciences PharMingen (San Diego, CA) and Upstate Biotechnology (Lake Placid, NY), respectively. Sulforhodamine B (SRB), taxol, vincristine, carboxyfluorescein succinimidyl ester (CFSE), 4,6-diamidino-2-phenylindole dihydrochloride (DAPI), propidium iodide (PI) and all other chemical compounds were obtained from Sigma-Aldrich (St. Louis, MO). KUD773 (Figure 1A) was synthesized with the purity of more than 98% by the examination using ^1^H and ^13^C NMR and elemental analysis (C, H, N, S) [22].

### Tissue explants and cell culture

All human tissue samples were obtained following informed consent of the donors and after full review by the Ethics Review Committee at National Taiwan University Hospital. Human prostate specimens were from males by transurethral resection of the prostate in National Taiwan University Hospital. All patients with prostatism histories were diagnosed to have benign prostate hyperplasia by rectal digital examination, transrectal sonography of prostate and urodynamic studies. Isolation of human prostatic cells from prostatic tissue explants was described in the previous study [23]. The HRPC cell lines, PC-3 and DU-145, were obtained from American Type Culture Collection (Rockville, MD). The cells were cultured in RPMI1640 medium with 10% FBS (v/v) and penicillin (100 units/ml)/streptomycin (100 μg/ml). Cultures were maintained in a humidified incubator at 37°C in 5% CO_2_/95% air.

### SRB assay

Cells were seeded in 96-well plates in medium with 5% FBS. After 24 h, cells were fixed with 10% trichloroacetic acid (TCA) to represent cell population at the time of compound addition (T_0_). After additional incubation of 0.1% dimethylsulfoxide (DMSO) or KUD773 for 48 h, cells were fixed with 10% TCA and SRB at 0.4% (w/v) in 1% acetic acid was added to stain cells. Unbound SRB was washed out by 1% acetic acid. SRB bound cells were solubilized with 10 mM Trizma base. The absorbance was read at a wavelength of 515 nm. Using the following absorbance measurements, such as time zero (T_0_), control growth (C), and cell growth in the presence of KUD773 (Tx), the percentage growth was calculated at each of the compound concentrations levels. Percentage growth inhibition was calculated as: [1- (Tx-T_0_)/(C-T_0_)] × 100%. Growth inhibition of 50% (GI_50_) is determined at the compound concentration which results in 50% reduction of total protein increase in control cells during the compound incubation [24].

### Cell proliferation assay with CFSE labeling

The cells were adjusted to a density of 10^6^ cells/ml and were treated with CFSE at a final concentration of 10 μM. After incubation at 37°C for 10 min, labeling was blocked by the addition of RPMI medium with 10% FBS. Tubes were placed in ice for 5 min and then washed. After centrifugation, the cells were seeded in RPMI medium with 10% FBS for 24, 48 and 72 h at 37°C under 5% CO_2_/95% air. After the treatment, the fluorescence intensity was determined by flow cytometric analysis.

### Flow cytometric analysis of PI staining

After the treatment of cells with vehicle (0.1% DMSO) or KUD773 for the indicated times, the cells were harvested by trypsinization, fixed with 70% (v/v) alcohol at 4°C for 30 min and washed with phosphate-buffered saline (PBS). After centrifugation, cells were incubated in 0.1 M of phosphate-citric acid buffer (0.2 M NaHPO_4_, 0.1 M citric acid, pH7.8) for 30 min at room temperature. Then, the cells were centrifuged and resuspended with 0.5 ml PI solution containing Triton X-100 (0.1% v/v), RNase (100 μg/ml) and PI (80 μg/ml). DNA content was analyzed with the FACScan and CellQuest software (Becton Dickinson, Mountain View, CA).

### Western blotting

After the indicated exposure time to DMSO or KUD773, the cells were washed twice with ice-cold PBS and reaction was terminated by the addition of 100 μl ice-cold lysis buffer (10 mM Tris–HCl, pH 7.4, 150 mM NaCl, 1 mM EGTA, 1 mM phenylmethanesulfonyl fluoride (PMSF), 10 μg/ml aprotinin, 10 μg/ml leupeptin, and 1% Triton X-100). For western blot analysis, the amount of proteins (40 μg) were separated by electrophoresis in a 10% polyacrylamide gel and transferred to a nitrocellulose membrane. After an overnight incubation at 4°C in PBS/5% nonfat milk, the membrane was washed with PBS/0.1% Tween 20 for 1 h and immuno-reacted with the indicated antibody for 2 h at room temperature. After four washings with PBS/0.1% Tween 20, the anti-mouse or anti-rabbit IgG (dilute 1:2000) was applied to the membranes for 1 h at room temperature. The membranes were washed with PBS/0.1% Tween 20 for 1 h and the detection of signal was performed with an enhanced chemiluminescence detection kit (Amersham).

### Microtubule assembly assay

After the treatment with the indicated agent for 24 h, the cells were harvested by trypsinization and collected by centrifugation. The cells were lysed with 0.1 ml of hypotonic buffer (1 mM MgCl_2_, 2 mM EGTA, 0.5% NP-40, 2 mM PMSF, 200 U/ml aprotinin, 100 μg/ml soybean trypsin inhibitor, 5.0 mM ε-amino caproic acid, 1 mM bezamidine and 20 mM Tris–HCl, pH 6.8). The cytosolic and cytoskeletal fraction of cell lysate were separated by centrifugation at 16,000×g for 15 min. The supernatant contained cytosolic tubulin. The pellet representing the particulate fraction of polymerized tubulin was resuspended in 0.1 ml hypotonic buffer. Tubulin contents in both fractions were detected by Western blotting.

### Confocal immunofluorescence microscopic examination

Cells were seeded in 8-well chamber slides. After the compound treatment for 12 h, the cells were fixed with 100% methanol at −20°C for 5 min and incubated in 1% bovine serum albumin (BSA) containing 0.1% Triton X-100 at 37°C for 30 min. The cells were washed twice with PBS for 5 min and stained with anti-β-tubulin or anti-γ-tubulin antibody at 37°C for 1 h and then, the FITC-conjugated secondary antibody at 37°C for 40 min. Nuclear staining was performed by 1 μg/ml DAPI. The cells were analyzed by a confocal laser microscopic system (Leica TCS SP2).

### Aurora A luminescent kinase assay

Test compounds, enzyme and substrate-tetra (LRRWSLG)/dithiothreitol (DTT)/ATP mix were dissolved in Aur assay buffer (50 mM Tris–HCl pH 7.4, 10 mM NaCl, 10 mM MgCl_2_,100 μg/ml BSA) separately before the assay. Twenty five μl of test compounds in DMSO stock solution and 10 μl enzyme were added into 96 well U-bottomed plate (268152, NUNC, Rochester, NY), and incubated at 25°C for 15 min. Fifteen μl substrate/DTT/ATP mix was added into the plate to initiate the assay. The assays was carried out at 37°C for 90 min in a final volume of 50 μl including the following components: 50 mM Tris–HCl pH 7.4, 10 mM NaCl, 10 mM MgCl_2_,100 μg/ml BSA, 1 mM DTT, 15 μM peptide substrate, 5 μM ATP, 90 ng/well Aurora A kinase, and test compound. A volume (50 μl) of Kinase-Glo Plus Reagent (Promega) was added into the completed kinase reaction, followed by incubation at 25°C for 20 min. Seventy μl of reaction mixture was transferred to 96 well black plate and luminescence was recorded by vector2 V (1420 multilable HTS counter, Perkin Elmer, Shelton, CT).

### Data analysis

Data are presented as the mean ± SEM for the indicated number of separate experiments. Student’s *t*-test is applied for comparison of two groups. *P*-values less than 0.05 are statistically considered significant.

## Results

### Determination of anti-proliferative activity of KUD773

The effect of KUD773 on cell proliferation was examined using SRB assay based on the measurement of cellular protein content. The data demonstrated that KUD773 exhibited a higher activity against prostate cancer PC-3 (GI_50_ = 1.7 μM) and DU-145 (GI_50_ = 3.2 μM) than primary human prostate cells (GI_50_ = 18.3 μM) (Figure 1B). The anti-proliferative activity was further confirmed by flow cytometric analysis of CFSE-staining assay. After the conjugation with cellular proteins, CFSE labeling is allocated evenly to daughter cells after cell division. The fluorescence intensity is decreased thereafter. The data in Figure 1C demonstrated a doubling time of 30.2 h in vehicle-treated PC-3 cells. The exposure of KUD773 (3 μM) significantly inhibited the decrease of fluorescence intensity showing a doubling time of 53.4 h (Figure 1C).

### Effect of KUD773 on the progression of cell cycle in PC-3 cells

**Figure 2:**
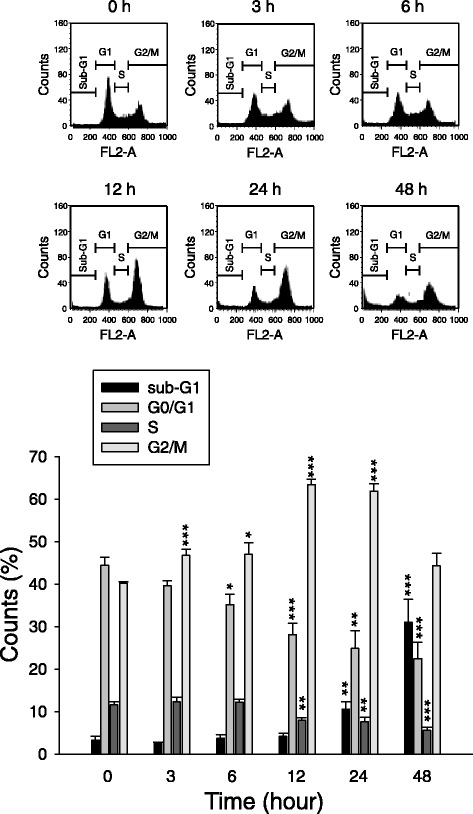
**Effect of KUD773 on cell cycle progression and apoptosis.** PC-3 cells were treated without or with KUD773 (3 μM) for the indicated times. Then, the cells were fixed and stained with PI to analyze DNA content by flow cytometric analysis. Data are expressed as mean ± SEM of three determinations. *P < 0.05, **P < 0.01 and ***P < 0.001 compared with the zero time control.

**Figure 3:**
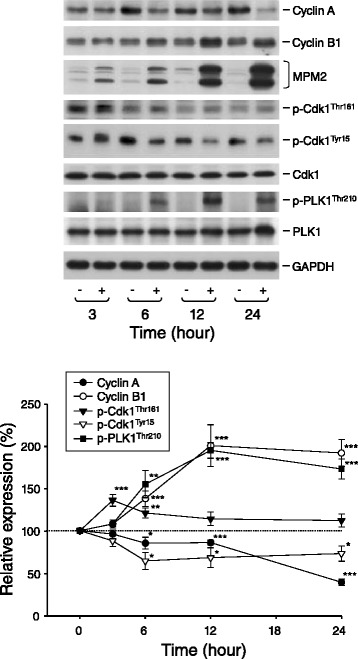
**Effect of KUD773 on the expressions of several cell-cycle regulators.** PC-3 cells were incubated in the absence or presence of KUD773 (3 μM) for various times. Cells were harvested and lysed for the detection of the indicated protein expression by Western blot. The expression was quantified using the computerized image analysis system ImageQuant (Amersham Biosciences). The images are representative of three independent experiments. The data are expressed as mean ± SEM of three to four independent experiments. *P < 0.05, **P < 0.01 and ***P < 0.001 compared with the control of 100%.

**Figure 4:**
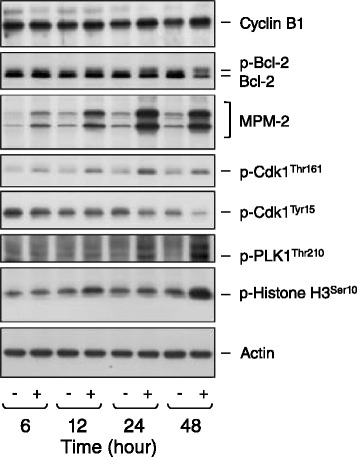
**Effect of KUD773 on the expression of several proteins in DU-145 cells.** The cells were incubated in the absence or presence of KUD773 (3 μM) for various times. Cells were harvested and lysed for the detection of the indicated protein expression by Western blot. The images are representative of two independent experiments.

Cellular stresses may cause the arrest of cell cycle at particular phase, leading to the inhibition of cell proliferation. The effect of KUD773 on the progression of cell cycle was examined by flow cytometric analysis of PI staining with DNA. As a consequence, KUD773 induced an arrest of the cell cycle at G2/M phase in a time-dependent fashion and a subsequent increase of hypodiploid sub-G1 phase (apoptosis) (Figure 2). The progression of cell cycle is regulated by cyclins and the binding partner Cdks. Cyclin A/Cdk2 complex activity tightly regulates S phase and G2 phase. In contrast, cyclin B1/Cdk1 complex controls the progression from G2- to M-phase [25]. The exposure of cells to KUD773 resulted in down-regulation of cyclin A while up-regulation of cyclin B1 in PC-3 cells (Figure 3). The effect was associated with an increased phosphorylation of Cdk1 at Thr^161^ (a stimulatory phosphorylation site) and a concomitant decreased phosphorylation at Tyr^15^ (an inhibitory phosphorylation site) (Figure 3), suggesting the activation of Cdk1 [26]. Additionally, the detection of a dramatic increase of mitotic phosphoproteins using antibody to MPM-2 showed that KUD773 induced a mitotic arrest of the cell cycle in PC-3 cells (Figure 3). PLK1 activity, another cell cycle regulator, is altered at different mitotic stages through the phosphorylation at Thr^210^. The phosphorylation peaks in prometaphase and gradually disappears in anaphase [27]. KUD773 maintained a high level of PLK1 phosphorylation at Thr^210^ in PC-3 cells (Figure 3) indicating that KUD773 induced the mitotic arrest at prometaphase to metaphase. Similar data were also detected in DU-145 cells (Figure 4).

### Effect of KUD773 on inhibition of microtubule assembly and formation of monopolar spindle

**Figure 5:**
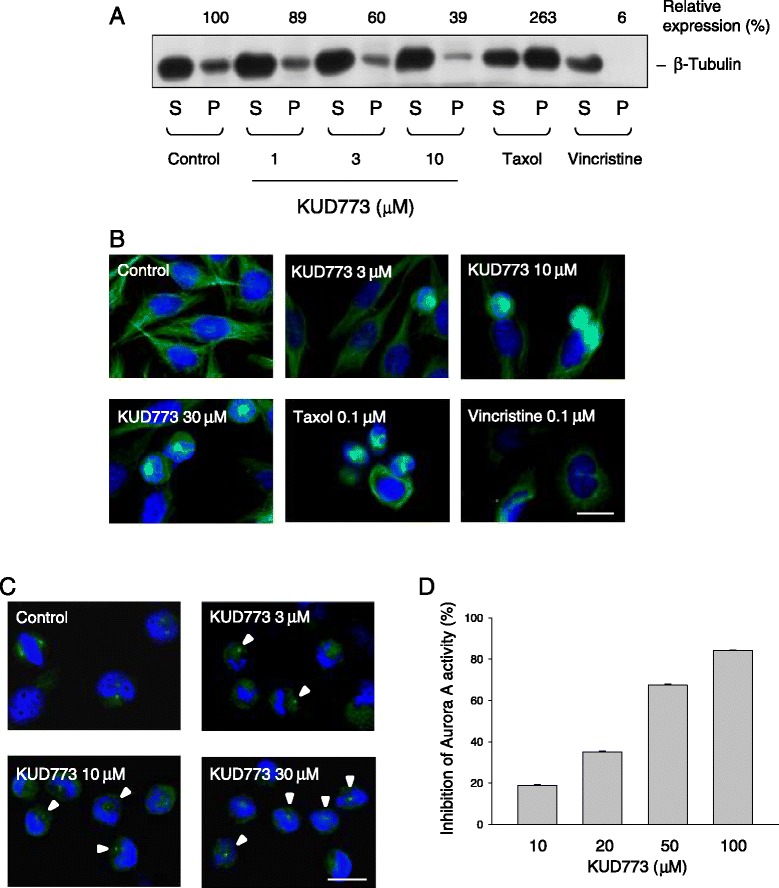
**Effect of KUD773 on microtubule assembly assay. (A)** PC-3 cells were incubated in the absence or presence of the indicated agent (taxol, 0.1 μM; vincristine, 0.1 μM). The cells were harvested and separated into soluble (S, microtubule disassembly) and particulate form (P, microtubule assembly), and proteins were detected by Western blot analysis. The data for relative expression of particulate fraction are also demonstrated. **(B)** Cells were incubated with or without the indicated agent for 12 h. The cells were fixed for the confocal microscopic detection of microtubule organization by staining with anti-β-tubulin antibody (green fluorescence) and DAPI (blue fluorescence, for nuclear detection). **(C)** Cells were treated in the same condition to (B) but the detection of microtubule organization by staining with anti-γ-tubulin antibody (green fluorescence) and DAPI (blue fluorescence). Arrowhead, monopolar γ-tubulin staining. *Scale bar*, 20 μm. **(D)** Aurora A luminescent kinase assay was performed. The data are expressed as mean ± SEM of three independent experiments.

Several cellular stresses, including disturbance of microtubule dynamics and inhibition of Aurora A during mitosis, have been suggested to induce mitotic arrest of the cell cycle [28,29]. To determine whether tubulin/microtubule served as a target for KUD773, the microtubule assembly assay was performed which the assembly and disassembly microtubules in particulate and soluble fractions, respectively, were separated. As a result, the microtubule assembly (particulate fraction) was decreased in the presence of KUD773 and vincristine (positive control). In contrast, taxol increased the microtubule assembly (Figure 5A). Interestingly, KUD773 induced the formation of monopolar spindle with 12.7 ± 1.1%, 37.8 ± 6.2% and 51.7 ± 3.0%, respectively, at 3, 10 and 30 μM (Figure 5B). The effective concentration for 50% formation (EC_50_) was 26.2 μM. The data of γ-tubulin staining also showed the formation of monopolar spindle (Figure 5C). Several lines of evidence reveal that the deficiency of Aurora A activity leads to the failure in centrosomal duplication or centrosomal separation, producing a monopolar spindle array [30,31]. In this study, Aurora A luminescent kinase assay was performed. The data showed that KUD773 inhibited Aurora A in a concentration-dependent manner with an IC_50_ of 30.0 μM (Figure 5D), which was similar to the induction of monopolar spindle formation, indicating that the anti-Aurora A activity might partly contribute to the formation of monopolar spindle.

### Effect of KUD773 on the expression of Bcl-2 family proteins and caspase cascades

**Figure 6:**
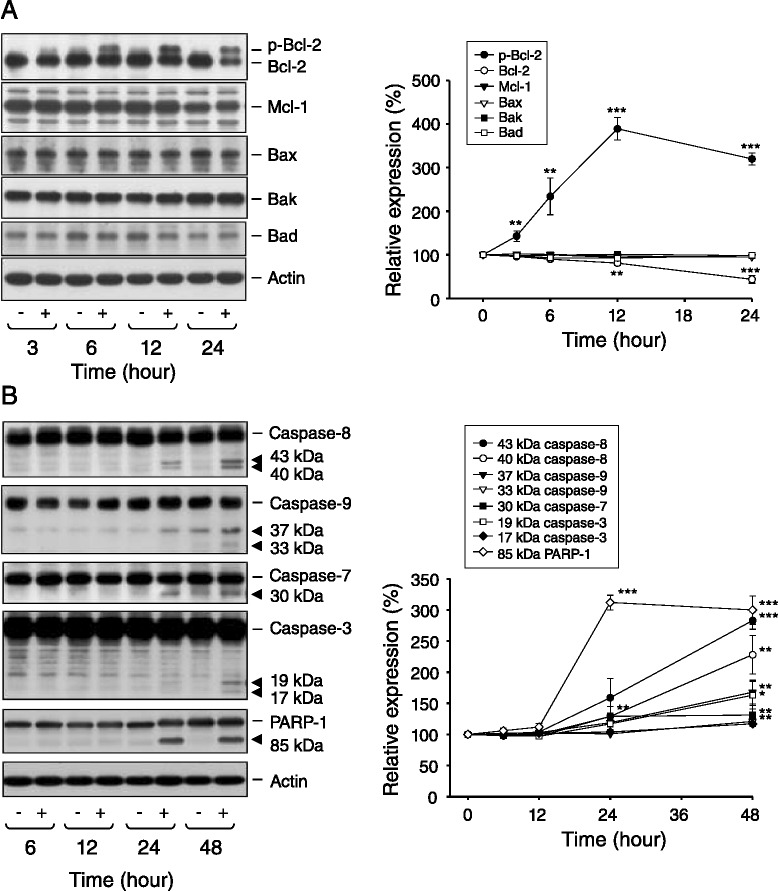
**Effect of KUD773 on the expression of Bcl-2 family members and caspases. (A and B)** PC-3 cells were incubated in the absence or presence of KUD773 (3 μM) for various times. Cells were harvested and lysed for the detection of the indicated protein expression by Western blot. The expression was quantified using the computerized image analysis system ImageQuant (Amersham Biosciences). The data are expressed as mean ± SEM of three to four independent experiments. *P < 0.05, **P < 0.01 and ***P < 0.001 compared with the control of 100%.

Bcl-2 family proteins play a central role on regulating the integrity of mitochondrial membrane and caspase-mediated apoptotic cell death. Several pro-apoptotic members in this family, including Bax, Bak and Bad, promote the release of cytochrome *c* and other proteins through the outer mitochondrial membrane. Some members, such as Bcl-2 and Mcl-1, are able to antagonize pro-apoptotic members and to keep mitochondrial integrity [32,33]. In this regard, KUD773 induced a profound increase of Bcl-2 phosphorylation followed by a significant decrease of Bcl-2 protein expression in PC-3 cells. In contrast, KUD773 did not modify the protein levels of the other members, including Mcl-1, Bax, Bak and Bad (Figure 6A). Bcl-2 phosphorylation, a biochemical marker for tubulin-targeting agents [34], also supported that KUD773 acted on tubulin for the execution of anticancer function.

Next, the activation of caspase proteases was examined by Western immunoblot analysis. Caspases were synthesized primarily as inactive precursors. As demonstrated in Figure 6B, the caspases were present as un-cleaved forms in untreated cells. After the exposure to KUD773, the cleavage of inactive precursors to active fragments was detected, in particular, at the 24-h and 48-h treatment. The cleavage of PARP-1, a preferred substrate for caspase-3 and caspase-7, was also apparent to KUD773 action (Figure 6B). The data reveal that the caspase activation is responsible for KUD773-mediated apoptotic cell death.

### Effect of KUD773 on the expression levels of MMP-2 and MMP-9

**Figure 7:**
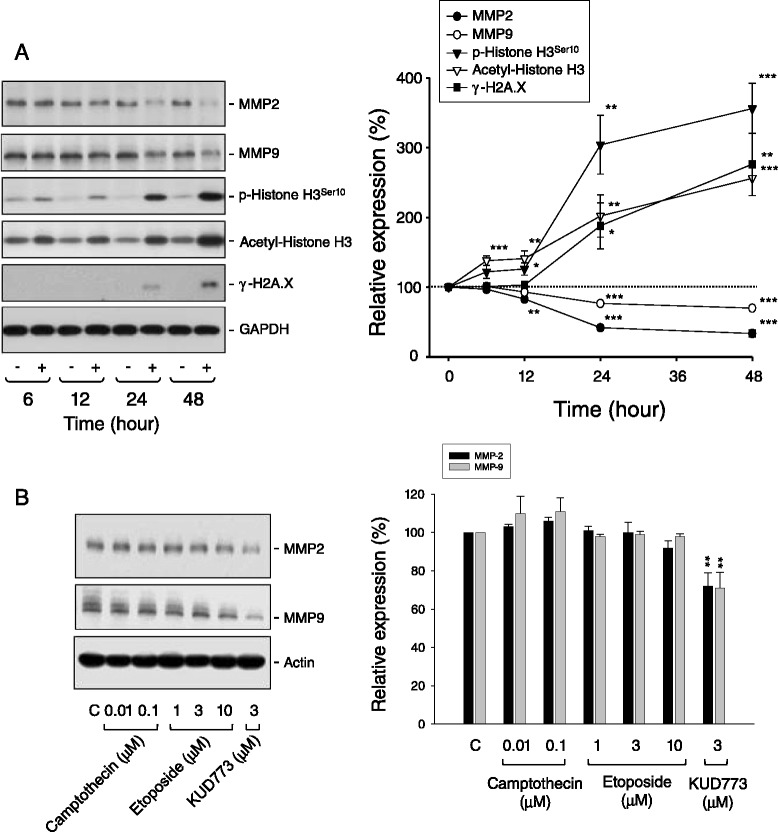
**Effect of KUD773 on MMP expression and histone H3 acetylation and phosphorylation.** PC-3 cells were incubated in the absence or presence of KUD773 (3 μM) for various times **(A)** or the indicated agent for 24 h **(B)**. Cells were harvested and lysed for the detection of the indicated protein expression by Western blot. The expression was quantified using the computerized image analysis system ImageQuant (Amersham Biosciences). The data are expressed as mean ± SEM of three independent experiments. *P < 0.05, **P < 0.01 and ***P < 0.001 compared with the control of 100%.

MMP-2 (gelatinase A) and MMP-9 (gelatinase B) are cancer-associated zinc-dependent endopeptidases. Both gelatinases play a central role in regulating cell migration, invasion and angiogenesis through cleavage of downstream substrates, including growth factors and their receptors, extracellular matrix and cytokines [35]. Accordingly, development of inhibitors against MMP-2 and MMP-9 is a potential anticancer strategy. In this study, KUD773 induced a time-dependent down-regulation of MMP-2 and MMP-9 expression. The effect was time-correlated with the increased phosphorylation and acetylation of histone H3 (two responses in mitosis), but was prior to the production of γ-H2A.X (a DNA damage response) (Figure 7A). The data indicated that the decreased expression of MMP-2 and MMP-9 was related to the mechanism of mitotic arrest but not through the DNA-damaged cell death because both camptothecin and etoposide (two topoisomerase poisons) which could induce DNA damage and cell death did not result in the down-regulation of MMP-2 and MMP-9 (Figure 7B).

## Discussion

Tubulin-targeting agents have long been used for clinical cancer chemotherapy against a wide variety of cancers. However, the mechanism-related toxicity, such as peripheral neuropathy, limits the therapeutic use of these agents [3,6]. The present study seeks to solve the problem by developing novel agents of introducing anti-Aurora A activity into anti-tubulin agents. The rationale is based on the strategy that mitotic interrupting agents always display high selectivity between cancer cells and normal cells [3,4,9,36]. Both tubulin-targeting and anti-Aurora A agents are mitotic interrupting agents. Furthermore, dual-effect inhibitors may reserve individual activities while decrease the toxicity because of the reduced concentrations used for reaching similar efficacy. The flow cytometric analysis of PI staining showed that KUD773 induced G2/M arrest of the cell cycle and a subsequent cell apoptosis. The data together with the dramatic increase of mitotic protein phosphorylation indicated the mitotic arrest other than G2 arrest to KUD773 action. The cell-based microtubule assembly assay showed that KUD773, similar to vincristine, inhibited tubulin polymerization and microtubule assembly. The SRB assay also has been performed to examine the effect of KUD773 on anti-proliferation in several cancer cell lines. The GI_50_ values were 2.2, 2.3, 2.1 and 5.2 in MDA-MB-231 (breast cancer), A549 (non-small cell lung cancer), HCT-116 (colorectal cancer) and HT-29 (colorectal cancer), respectively. It is not surprising that KUD773 is effective against all tested cancer cell lines because the action target on tubulin is universal in cells, in particular in proliferating cancer cells.

KUD773 induced a significant increase of Bcl-2 phosphorylation, a specific hallmark to tubulin-targeting agents [37]. It has been suggested that phosphorylated Bcl-2 fails to dimerize with Bax, leading to augmented levels of free Bax and acceleration of cell apoptosis [38]. Several studies show that Bcl-2 may bind Raf-1. Furthermore, Raf-1 depletion by geldanamycin inhibits Bcl-2 phosphorylation [37,39,40], suggesting that Raf-1 is a potential candidate for Bcl-2 phosphorylation. However, the responsible kinase for KUD773-mediated Bcl-2 phosphorylation needs further investigation.

Cdk1 interacts with cyclin B1 to form a heterodimer complex. The complex activity is required for the progression of cell cycle to mitotic prophase and metaphase. Moreover, it has been identified that Cdk1 activity is responsible for triggering cell death in certain signaling cascades under apoptotic stimuli, including exposure to tubulin-targeting agents [9,36,37]. The entry of cell cycle into mitosis is controlled by Cdk1 activation through several steps, including cyclin B1 binding and Cdk1 phosphorylation at Thr^161^. Moreover, dephosphorylation of Cdk1 at Tyr^15^ appears to be a critical step for Cdk1 activation during the progression into cell mitosis [25,41]. The data showed that KUD773 induced the activation of Cdk1 and mitotic arrest in both PC-3 and DU-145 cells. These events could guarantee the activation of apoptotic program because it has been extensively identified that premature activation of Cdk1 can result in mitotic catastrophe after a wide variety of cellular stresses [9,26,42].

The Aurora kinases, including Aurora A, Aurora B and Aurora C, are serine/threonine kinases that are implicated with critical processes in cell mitosis [7]. Aurora A, an oncogenic Aurora kinase, is mainly centrosomal and localizes to mitotic spindle. It is necessary for centrosome separation and microtubule assembly [8,9]. Aurora B has been shown to colocalize with end-binding protein 1 on central spindle during anaphase and on midbody during cytokinesis, playing a crucial role in chromosome biorientation, cytokinesis and spindle assembly checkpoint [43,44]. By contrast, much less is known about the cellular function of Aurora C. However, recent studies show that overexpression of active Aurora C in NIH-3T3 stable cell lines leads to tumor formation when injected into nude mice, showing an oncogenic activity of active Aurora C [45]. The present data demonstrated that KUD773 displayed an inhibitory activity against Aurora A using luminescent kinase assay. The inhibitory activity was further validated by the observation that KUD773 induced the formation of monopolar spindle because Aurora A was responsible for centrosome separation. The effect was similar to other Aurora A inhibitors, such as VX-680 that led to monopolar spindle formation in a wide variety of cancer cells [46]. Of note, KUD773 caused histone H3 phosphorylation at Ser^10^. It has been documented that both Aurora A and Aurora B are able to phosphorylate histone H3 [47]. However, Aurora B is specifically responsible for the phosphorylation of histone H3 not only at Ser^10^ but also at Ser^28^ that contribute to mitotic chromosome condensation [44,47,48]. KUD773 showed effective inhibition on Aurora A but not Aurora B (data not shown), explaining that histone H3 phosphorylation still occurred during KUD773-induced mitotic arrest.

MMPs, zinc-dependent endopeptidases, are capable of degrading a wide spectrum of extracellular matrix proteins. MMPs are also involved in proteolysis of many non-matrix substrates, such as cytokines and chemokines [49]. Recently, MMP activity has been known to play a key role in the cleavage of Fas, leading to an increased resistance to FasL-mediated apoptosis in various cancer cells [50]. MMPs are, therefore, thought to play a crucial role on directing several cell functions, such as cell migration, proliferation, differentiation, apoptosis and angiogenesis [35,49]. Among this family of members, MMP-2 and MMP-9 have been characterized as crucial factors contributing to angiogenesis and tumor invasion [35]. Moreover, highly metastatic variants of prostate cancers are revealed to contain relatively high levels of MMP-2 and MMP-9 [51]. The present study demonstrated that KUD773 induced a significant down-regulation of MMP-2 and MMP-9 expression, suggesting an anti-metastatic potential of KUD773 although this effect has not been determined in this study. The data also revealed that the inhibitory effect on MMP expression levels was related to KUD773-mediated anti-mitotic activity. It has been suggested that inhibition of tubulin polymerization may cause the suppression of genetic transcription and mRNA instability of both MMP-2 and MMP-9 [52]. It may explain KUD773-induced down-regulation of these two MMP members. However, the underlying mechanism warrants further investigation.

## Conclusions

The data suggest that KUD773 induces apoptotic signaling in a sequential manner. It inhibits tubulin polymerization associated with an anti-Aurora A activity, leading to Cdk1 activation and mitotic arrest of the cell cycle that *in turn* induces Bcl-2 degradation and a subsequent caspase activation in HRPCs. The inhibitory activity on the expression of MMP-2 and MMP-9 may add the anticancer potential of KUD773. Because the class of compounds with imidazole or thiazole structure has been reported to display some other activities, such as inhibition of Akt, p38 MAPK and tyrosine kinase, the anticancer effect of KUD773 may also attributed to other undefined mechanisms that warrant further elucidation. However, KUD773 which displays both anti-tubulin and anti-Aurora A activities is a novel design for dual-effect inhibitors although its structure needs further optimization for activity improvement.
